# The impact of aesthetic education on university students’ psychological wellbeing: exploring mediating and moderating effects

**DOI:** 10.3389/fpsyg.2025.1515671

**Published:** 2025-03-19

**Authors:** Lan Ye, Yanwei Li, Na Zhang

**Affiliations:** ^1^College of Cabin Crew, Civil Aviation University of China, Tianjin, China; ^2^School of Economics and Management, Civil Aviation University of China, Tianjin, China; ^3^School of Economics and Management, Beijing Information Science and Technology University, Beijing, China

**Keywords:** psychological wellbeing, basic psychological needs, aesthetic education, self-determination theory, college students

## Abstract

With rising psychological concerns among college students, educators and scholars are increasingly emphasizing mental health. As a key component of university curricula, aesthetic education plays a significant role in enhancing the psychological wellbeing of college students. Grounded in Self-Determination Theory (SDT), which focuses on three fundamental psychological needs—autonomy, competence, and relatedness—this study explores how aesthetic education influences the psychological well-being of university students. Specifically, aesthetic education fosters autonomy through self-expression, enhances competence by promoting creative development, and nurtures relatedness by facilitating social interaction in collaborative artistic activities. The study examines the mediating role of basic psychological needs and the moderating effects of gender and age. A total of 513 college students from China were surveyed using convenience sampling. Descriptive statistics, correlation analysis, hierarchical regression and mediation analysis were conducted using SPSS 26.0 to assess key variables, including aesthetic education, basic psychological needs, and psychological well-being. The results reveal significant positive correlations between aesthetic education, students’ psychological wellbeing, and the fulfillment of basic psychological needs. Furthermore, aesthetic education significantly predicts the psychological well-being, with the satisfaction of basic psychological needs partially mediating this relationship. Gender and age were found to moderate the relationship between aesthetic education and psychological wellbeing to varying extents. These findings elucidate important associational pathways between aesthetic education, college students’ psychological wellbeing, and the satisfaction of basic psychological needs, contributing both to the understanding of SDT and to practical applications in the context of university-level aesthetic education.

## 1 Introduction

In recent years, the psychological well-being of university students has garnered increasing attention (Hanawi et al., 2020). Many students face challenges related to anxiety, depression, and stress management, often arising from academic pressures, competitiveness in the job market, and difficulties in navigating social relationships (Alotaibi et al., 2022; Morales-Rodríguez et al., 2020). These stressors not only affect students’ academic performance but also have a profound impact on their social and psychological wellbeing. Education can significantly influences students’ mental health, potentially affecting their overall growth and quality of life (Halpern-Manners et al., 2016).

Among various educational approaches, aesthetic education has emerged as a significant strategy, gaining increasing attention from both educators and scholars. Aesthetic education is defined as the ability to cultivate an idealized perception of beauty, as well as the capacity to shape aesthetic behavior through the principles of beauty (Lee and Chao, 2023). University students require time to develop and refine their aesthetic awareness, attitudes, and behaviors. This form of education emphasizes the enjoyment and understanding of beauty and art, which not only enhances academic performance but also contributes to emotional and psychological well-being (Ashurova, 2021). Aesthetic education can help students cultivate their aesthetic sensibilities, improve emotional expression and regulation, thereby alleviating psychological stress and promoting mental health. Consequently, investigating the impact of aesthetic education on the psychological well-being of university students has become a critical area of contemporary educational research.

Numerous studies have highlighted the significant benefits of aesthetic education for university students’ psychological well-being and personal development (Lee and Chao, 2023). According to Adekunle (2023), aesthetic education not only influences university students’ cognitive abilities but also plays a crucial role in enhancing their emotional and social well-being. This form of education can shape students’ emotions and behaviors while simultaneously fostering the growth of their personality and temperament (Lee and Chao, 2023). University students exist within a complex environment that encompasses physical, emotional, mental, and spiritual dimensions, and they acquire cognitive skills through various academic disciplines. However, subjects such as mathematics, chemistry, and foreign languages often fail to address students’ emotional and spiritual needs. From this perspective, aesthetic education serves as a fundamental source of sensitivity, profoundly influencing students’ personal growth (Mercin and Alaku, 2007). Aesthetic education goes beyond the cultivation of creative abilities; it nurtures an appreciation for beauty, sharpens observational skills, and fosters a deep understanding of the various forms that enrich university students’ overall experiences (Dickson et al., 2022). Consequently, aesthetic education can be considered one of the most effective pedagogical approaches, engaging not only the intellect but also the senses, emotions, and imagination, which may allow for holistic growth beyond standard academic metrics (Ma et al., 2021). Despite the widespread promotion of aesthetic education as a vital source of knowledge in China, it remains underappreciated by the general public (Yue, 2022).

This research aims to address three key objectives. First, this study aims to examine the relationship between aesthetic education and university students’ psychological wellbeing. Exploring the association between the two can help explain how aesthetic education affects the psychological wellbeing of Chinese university students. Second, this study aim to apply Self-Determination Theory’s (SDT) by using the concept of psychological needs satisfaction as a mediating variable to explain the causal mechanisms through which aesthetic education impacts university students’ psychological wellbeing. According to SDT, the environment can either facilitate or hinder intrinsic motivation and the satisfaction of basic psychological needs (BPN), depending on how well it supports or undermines autonomy, competence, and relatedness (Deci and Ryan, 2000). Previous studies have demonstrated that educational environments that promote autonomy fulfill students’ psychological needs, thereby enhancing their academic motivation, engagement, and psychological well-being (Deci et al., 2001). In the context of aesthetic education, the way in which courses are designed and experienced can either support or inhibit the satisfaction of these psychological needs. When aesthetic education courses are structured to foster autonomy, competence, and relatedness, they can significantly enhance students’ psychological well-being. Third, this study investigates both gender and age as a moderating factors in the relationship between aesthetic education and its impact on college students’ psychological wellbeing. While aesthetic education fosters emotional expression, cognitive development, and overall life satisfaction in both male and female students, gender-specific factors might influence how these benefits are experienced and to what extent they are manifested. Additionally, age-related differences are likely to shape how students engage with and benefit from aesthetic education. For example, younger students may show greater emotional receptiveness and social engagement, while older students may derive more benefit from cognitive and self-regulation (Ryan and Deci, 2000). By understanding the diverse psychological needs of students across different age groups, educators can better tailor aesthetic education programs to support the satisfaction of basic psychological needs, optimizing the effectiveness of these programs for all participants.

## 2 Theory and hypotheses

### 2.1 Aesthetic education

The term “aesthetic” is derived from “estesio,” meaning “I feel beauty.” Aesthetic involves the study and appreciation of taste and beauty, which are experienced through sensory and emotional responses to values, nature, beauty, taste, and the arts (D'Olimpio, 2022). It encompasses the examination of beauty and art, as well as our perception of “beauty” in the world around us (Lee and Chao, 2023). A sense or appreciation of beauty can be cultivated through education and experience. Moreover, learning about beauty and life experiences can alter one’s perspective and evaluation of beauty (Yu, 2013). Crawford (1991) defined aesthetic as “the branch of philosophical inquiry that involves the critical reflection on our experience and evaluation of art.” Greene (2018) described aesthetic as: “An intentional undertaking designed to nurture appreciative, reflective, cultural, participatory engagements with the arts by enabling learners to notice what is there to be noticed, and to lend works of art their lives in such a way that they can achieve them as variously meaningful.” In summary, knowledge of aesthetic encompasses a broad spectrum of intellectual and sensory literacies.

Aesthetic education is defined as a purposeful effort to foster appreciation, reflection, cultural understanding, and participatory engagement with the arts and creative works (Greene, 2001; D'Olimpio, 2022). These aesthetic experiences enable learners to become attuned to color, patterns, and tastes, encouraging them to perceive and reason in novel ways. According to Greene (2001), aesthetic education plays a crucial role in the cognitive, perceptual, emotional, and creative development of learners, which continues to be significant throughout higher education and plays a vital role in university students’ holistic growth (Adekunle, 2023). Consequently, aesthetic education has a broader scope than commonly perceived, teaching individuals to perceive natural and social beauty in ideal ways. Moreover, it develops individuals’ ability to change the reality of aesthetic activity in alignment with principles of beauty. Acquiring and refining one’s aesthetic awareness, attitudes, and actions is a gradual process, shaped by age and socioeconomic factors, which may influence the various stages and depths of this development. Additionally, aesthetic education is rooted in an individual’s assimilation of societal aesthetic culture, taking on several forms across different contexts (Lee and Chao, 2023).

The primary goal of education is to cultivate individuals who understand their living conditions, recognize their social responsibilities, embrace civic consciousness, and acknowledge the importance of active participation in societal service (Adekunle, 2023). Aesthetic education serves as one of the most effective means of conveying human ideas, culture, identity, lifestyles, emotions, and societal experiences (Zeb et al., 2021). It is an educational framework that utilizes the elements and attributes of beauty to stimulate learners’ senses through activities such as music, theater, visual arts, color, sound, taste, scent, and the direct experience of the environment and nature (Marshalsey, 2023; Furman, 2000). Consequently, aesthetic education is the most effective way to shape, nurture, develop, lead, and prepare the next generation of healthy individuals and communities (Jin and Ye, 2022). University is a period when young people encounter a wide range of challenges, including academic pressures, emotional struggles, financial stress, peer influences, and parental conflict—greatly impacts an individual’s psychological wellbeing. In this context, aesthetic education plays a vital role in addressing these challenges and promoting mental health (Jin and Ye, 2022).

### 2.2 Impact of aesthetic education on psychological wellbeing

Psychological wellbeing is often defined as a state that combines health, happiness, and prosperity. It involves not only feeling good but also functioning effectively in daily life situations (Elliott and Gramling, 1990). This concept is closely linked to both positive and negative affect, happiness, life satisfaction, creative thinking, prosocial behavior, and optimal physical health (Sheldon and Kasser, 1998; Diener and Biswas-Diener, 2008). Higher levels of psychological wellbeing have been associated with various physical and mental health benefits, including a more robust immune system, improved sleep quality, reduced blood pressure, and even greater longevity (Carr, 2004).

Psychological wellbeing is a fundamental aspect of university students’ overall health and academic success. It refers to a state of positive mental health that includes emotional regulation, life satisfaction, personal growth, purpose, and self-acceptance. This concept involves an individual’s ability to engage meaningfully with life, handle stress, and experience a sense of fulfillment (Ryff, 1989). Psychological wellbeing encompasses emotional, psychological, and social dimensions, including factors such as life satisfaction, stress management, and mental health (Oishi and Westgate, 2022). University students often face significant challenges that can adversely affect their psychological wellbeing, such as academic pressure, financial stress, and social adjustment (Kordzanganeh et al., 2021). Research has shown that psychological wellbeing is not only linked to academic performance but also to personal development and future career success (Diener and Sim, 2024).

Effective strategies to enhance psychological wellbeing among students include interventions aimed at reducing stress and promoting mental health awareness (Amanvermez et al., 2023). For example, programs focused on mindfulness and emotional regulation have been shown to improve students’ mental health outcomes and academic performance (Ameli et al., 2020). Additionally, fostering supportive social networks and providing access to mental health resources are crucial for supporting students’ psychological wellbeing (AlAzzam et al., 2021). Therefore, addressing psychological wellbeing through comprehensive support systems is essential for the success and overall health of university students.

Empirical research demonstrates the positive impact of aesthetic education on students’ health and wellbeing. Recent studies suggest that aesthetic education can enhance both physical and psychological well-being, serving as a therapeutic support for diverse groups, including teenagers, university students, the elderly, and vulnerable populations (Daykin et al., 2008; Todd et al., 2017; Thomson et al., 2018). Evidence indicates that participation in arts activities can improve overall wellbeing (Jensen and Torrissen, 2019). A review highlights the current understanding of the aesthetic, artistic, and creative contributions of dance to health and wellbeing (Chappell et al., 2021). Among the dimensions of aesthetic experience, cognitive synergies, elaboration, the paratelic mode, and expressive perception significantly and positively predict positive affect and well-being (Minmin and Mo, 2024). In higher education, aesthetic education serves as a platform for nurturing students’ emotional, cognitive, moral, and creative development. This underscores the importance of prioritizing aesthetic education in higher education institutions to cultivate students’ artistic and cultural awareness, enhance their creativity, and develop both practical skills and interdisciplinary talents—extending beyond the fine arts.

Similarly, art-based pedagogy emphasizes the integration of various art form (e.g., theater, visual art, painting, music) with other subjects to promote learning processes (Rieger and Chernomas, 2013). Previous studies has also demonstrated that students can utilize creative tools, artwork, or campaigns to showcase how artists foster healthy work habits and address mental health challenges (Siddins, 2021). Universities play a pivotal role in shaping students’ character and values, as well as in promoting their intellectual, physical, artistic, and professional development (Jing, 2014). Aesthetic education, by fostering emotional expression, critical thinking, and personal growth, plays a crucial role in enhancing students’ moral development and, consequently, contributes positively to their psychological well-being. Therefore, we propose the following hypothesis:

*H1*: Aesthetic education has a positive influence on psychological wellbeing.

### 2.3 Role of basic psychological needs as a mediator between aesthetic education and psychological wellbeing

The psychological wellbeing of university students is a multifaceted construct encompassing emotional, mental, and social dimensions of health (Ryff and Keyes, 1995). This concept is of paramount importance, as it directly influences students’ academic performance, personal development, and overall quality of life. Aesthetic education, which emphasizes the cultivation of an appreciation for beauty and engagement with diverse forms of art, has been shown to have a substantial impact on enhancing psychological wellbeing. Recent research has increasingly focused on understanding and improving the psychological wellbeing of university students, especially in light of the growing prevalence of mental health challenges his population in various global contexts, in Kuala Lumpur (Hanawi et al., 2020). SDT provides a robust theoretical framework for examining how aesthetic education may facilitate improvements in psychological wellbeing by addressing fundamental psychological needs.

According to Deci and Ryan (1987), SDT posits that the satisfaction of three core psychological needs—autonomy, competence, and relatedness—is essential for fostering intrinsic motivation and overall psychological well-being. Autonomy refers to the experience of making choices and feeling in control of one’s actions (Guay et al., 2000). Competence involves the ability to successfully engage with optimally challenging tasks and achieve desired outcomes Relatedness, on the other hand, encompasses the sense of connection and belonging with others, characterized by mutual respect, interdependence, and meaningful relationships that facilitate an individual’s need for social integration and interpersonal bonds (Deci and Ryan, 2000). Deci and Ryan (2000) argue that these three psychological needs are universal and intrinsic, with individuals naturally seeking environments that support their fulfillment. Consequently, a social context that nature and encourages the satisfaction of these three needs can enhance individual motivation and foster healthy psychological development.

Individuals are inherently motivated by the satisfaction of three key intrinsic psychological needs: autonomy, competence, and relatedness, which lead to the development and maintenance of intrinsic motivation and the integration of extrinsic motivation. Fostering intrinsic desires and promoting emotional regulation both depend on meeting these foundational psychological needs (Deci and Ryan, 2000). According to SDT, satisfying autonomy needs enables individuals to cultivate more autonomous forms of motivation, which in turn foster positive behaviors and attitudes (Deci and Ryan, 2000).

Research indicates that basic psychological needs play a critical role in education, particularly in the context of digital learning and educational technology. Hsu et al. (2019) provide empirical evidence supporting the application of a SDT-based model in online learning environments. Their findings suggest that the satisfaction of basic psychological needs fosters self-regulated motivation, which in turn is associated with higher perceived knowledge transfer and greater achievement of course objectives in online courses. Similarly, Huang et al. (2019) demonstrate that psychological needs and hedonic experiences are key factors in explaining the value of 3D virtual reality technology in educational settings, contributing to an overall enhancement of learners’ motivation and learning experiences. A recent study by Salikhova et al. (2020) highlights the scientific novelty of synthesizing and generalizing the application of SDT in the rapidly evolving field of digital education. Moreover, Shah et al. (2021) further corroborate these findings, suggesting that the satisfaction of basic psychological needs can stimulate students’ engagement within virtual learning environments.

Empirical evidence suggests that addressing these fundamental psychological needs through aesthetic education may contribute to improved psychological well-being (Jing and Ye, 2022). Activities that foster creativity and an appreciation for beauty within aesthetic education directly address these psychological needs, thereby contributing to improvements in students’ well-being (Deci et al., 2001). Aesthetic education may effectively satisfy these needs by providing students with opportunities to express themselves through creative activities (autonomy), develop their creative abilities (competence), and establish social connections through collaborative artistic projects (relatedness). Furthermore, engaging in artistic practices and receiving constructive feedback can bolster their sense of competence (Ryff et al., 2021), while participation in group art initiatives or art discussions fosters a sense of connection (Halverson and Sawyer, 2022).

Studies have demonstrated that environments that satisfy these fundamental psychological needs enhance emotional regulation, increase life satisfaction, and reduce stress levels (Deci et al., 2001; Vansteenkiste et al., 2004). Therefore, understanding the mediating role of these basic psychological needs offers valuable insights into the mechanisms through which aesthetic education enhances psychological well-being. Based on the aforementioned literature, this study propose the following hypothesis:

*H2*: Basic psychological needs mediate the relationship between aesthetic education and psychological wellbeing.

### 2.4 Role of age and gender as moderators between aesthetic education and psychological wellbeing

Aesthetic education, which fosters an appreciation for beauty and engagement with various forms of art, has been shown to significantly enhance psychological well-being (Jing and Ye, 2022). However, the impact of aesthetic education on psychological well-being may vary depending on demographic factors such as age and gender. Understanding these moderating effects can provide deeper insights into how aesthetic education influences individuals in distinct ways.

Age plays a crucial moderating role in shaping the psychological outcomes of c university students. Younger students, such as first- and second-year students, may experience greater benefits from aesthetic education due to their developmental stages, where creativity and emotional expression are particularly important. Arts-based programs, for example, can offer young students a platform for emotional expression, which is essential for their psychological development (Gibson and Ewing, 2020). Conversely, older university students may also derive significant benefits from aesthetic education, particularly in terms of cognitive and emotional engagement, which can alleviate feelings of loneliness and enhance overall life satisfaction (Dingle et al., 2021).

Moreover, gender differences can significantly moderate the effects of aesthetic education on psychological wellbeing. For females, increased engagement in arts-related activities may foster enhanced emotional expression and strengthen social connectedness, leading to more pronounced psychological benefits. In contrast, males may experience psychological benefits primarily through improved emotional regulation and stress reduction. These gender-based variations highlight the need to consider demographic factors when examining the impact of aesthetic education, forming the basis for the following hypotheses:

*H3*: Age moderates the relationship between aesthetic education and psychological well-being.

*H4*: Gender moderates the relationship between aesthetic education and psychological well-being.

## 3 Materials and methods

### 3.1 Participants and procedures

The survey was conducted at three universities in Mainland China, with a total of 513 students participants (130 men, 383 women) from Tianjin. Of the participants, 361 students (70.4%) were freshmen, 82 students (16.0%) were sophomores, and 66 students (12.9%) were juniors. Additionally, there were 2 seniors (0.4%) and 2 master’s students (0.4%). Participants’ ages were divided into age groups: 18–20, 21–22, 22–25, 25 and above. In terms of course enrollment, 261 students (50.9%) were required to take an arts education course, 82 students (16.0%) had it as an optional course, and 170 students (33.1%) had the option of either an elective or mandatory arts education course. The samples demographics are presented in Table 1.

**Table 1:** Demographic characteristics (*n* = 513).

Demographics	Classification	*n*	Percentage (%)	Cumulative percentage (%)
Age (years)	18–20	412	80.3	80.3
	21–22	97	18.9	99.2
	22–25	3	0.6	99.8
	>25	1	0.2	100.0
Gender	Male	130	25.3	25.3
	Female	383	74.7	100.0
Grade	Freshman	361	70.4	70.4
	Sophomore	82	16.0	86.4
	Junior	66	12.9	99.2
	Senior	2	0.4	99.6
	Master	2	0.4	100.0
Course	Required course	261	50.9	50.9
	Optional course	82	16.0	66.9
	Both	170	33.1	100.0

Based on their prior experiences and perceptions of the arts education course, students completed a questionnaire assessing their attitudes toward the course, the fulfillment of basic psychological needs, psychological well-being, and demographic information. The survey was administered online via the Wenjuanxing platform. Written informed consent was obtained from all participants, who were also made aware that they could discontinue the questionnaire at any point should they felt uncomfortable. Upon completion of the survey, participants were offered a small token of appreciation as compensation.

### 3.2 Ethical statement

Prior to conducting the investigation, ethical approval was obtained from the Ethics Committee for Biomedical Research of Medical College (No. 2023[K]030–20) at Hebei University of Engineering. Written informed consent was secured from all students, who were fully informed of their right to withdraw from the survey at any time without consequence.

### 3.3 Instruments

The items for the Aesthetic Education, Basic Psychological Needs, and Well-Being questionnaires were originally developed in English. For this study, all items were first translated into Chinese, followed by a back -translation into English to ensure cross-linguistic equivalence. No significant linguistic discrepancies were identified during this process (Brislin, 1970). All measurements were administered in Chinese.

### 3.4 Measures

#### 3.4.1 Aesthetic education

Aesthetic education was assessed using the Questionnaire on Perceptions of Aesthetic Education Courses (Lee and Chao, 2023). The original instrument was adapted to suit the needs of the university-level student population while preserving its core structure and focus on aesthetic education. Participants completed the questionnaires based on their perceptions of aesthetic education courses, using a 7-point Likert scale. The first factor, Aesthetic Appreciation (AEA), consisted of four items (e.g., “Musical activities enable students to gradually engage in music or drama-related creative works”) with a reliability of 0.96. The second factor, Aesthetic Implementation (AEI), also comprised four items (e.g., “Musical activities enable students to expand their art experience”) with a reliability of 0.97. The third factor, Aesthetic Evaluation (AEV), included four items (e.g., “Musical activities enable students to express their thoughts and feelings to others”) with a reliability of 0.97. The overall scale reliability for the study was 0.93.

#### 3.4.2 Basic psychological needs satisfaction

The satisfaction of students’ basic psychological needs was assessed using the Basic Psychological Needs Scale at Work (BPNS) (Deci et al., 2001; Ilardi et al., 1993; Kasser et al., 1992), employing a 7-point response scale. The first factor, autonomy, consisted of seven items (e.g., “I am feel free to express my ideas and opinions in the aesthetic education class.”) with a reliability coefficient of 0.77. The second factor, competence, included seven items (e.g., “People in the aesthetic education class tell me I am good at what I do.”) with a reliability coefficient of 0.75. The third factor, relatedness, also contained seven items (e.g., “People in the aesthetic education class care about me.”) with a reliability coefficient of 0.79. The overall scale reliability for this study was 0.92.

#### 3.4.3 Well-being

Students’ well-being was assessed using the Vitality Scale (Ryan and Frederick, 1997). The scale evaluates students’ general sense of well-being in life. A sample item is “I feel alive and vital.” Responses to the 7 items were rated on a 7-point scale, ranging from 1 (not at all true) to 7 (very true). The internal reliability of the Vitality Scale in this study was 0.84.

### 3.5 Results

#### 3.5.1 Descriptive statistics

Data analysis for this study was conducted using SPSS 26.0. Table 2 presents the distribution of students ‘preferences for different types of aesthetic education courses. The results reveal that 57.1% of students favored music courses, making them the most preferred form of aesthetic education. Dance and demonstration courses followed closely, with 48.1 and 39.0% of students expressing a preference for these, respectively. In contrast, literature-related aesthetic education courses- such as literature, Chinese traditional culture, art theory, and fine arts—were preferred by fewer than 30% of students.

**Table 2:** Demographic characteristics (*n* = 513).

Category	Like this lesson	Percentage (%)	Unlike this lesson	Percentage (%)
Music lesson	293	57.1	220	42.9
Fine arts lesson	151	29.4	362	70.6
Dance lesson	247	48.1	266	51.9
Demonstration lesson	200	39.0	313	61.6
Literature lesson	113	22.0	400	78.0
Chinese traditional culture lesson	132	25.7	381	74.3
Art theory lesson	119	23.2	394	76.8

Table 3 presents the means, standard deviations, and correlations of the key variables. Aesthetic education demonstrated a significant positive correlation with basic psychology needs (*r* = 0.48, *p* < 0.01) and well-being (*r* = 0.80, *p* < 0.01). Additionally, students’ satisfaction of basic psychology needs was positively correlated with their well-being (*r* = 0.57, *p* < 0.01). These findings provide support for Hypotheses 1.

**Table 3:** Descriptive statistics and correlation coefficient.

Variables	M	SD	1	2	3	4	5	6	7	8	9	10	11	12	13
1. Gender	1.75	0.44	1												
2. Age	1.21	0.43	−0.10*	1											
3. Education	1.45	0.78	−0.03	0.69**	1										
4. Course	1.82	0.90	−0.02	−0.00	0.04	1									
5. AEA	22.99	5.15	−0.08	0.04	0.01	−0.03	1								
6. AEI	23.29	4.94	−0.06	0.03	0.00	−0.03	0.96**	1							
7. AEV	23.21	5.00	−0.06	0.01	−0.01	−0.03	0.96**	0.97**	1						
8. AE	69.49	14.92	−0.07	0.02	0.00	−0.03	0.99**	0.99**	0.99**	1					
9. Autonomy	34.94	6.33	0.01	−0.02	−0.04	−0.08	0.44**	0.46**	0.46**	0.46**	1				
10. Competence	29.20	5.74	0.08	−0.03	−0.06	−0.01	0.33**	0.33**	0.34**	0.34**	0.84**	1			
11. Relatedness	42.08	7.42	0.04	−0.05	−0.06	−0.04	0.50**	0.53**	0.53**	0.52**	0.85**	0.82**	1		
12. Basic Psychology Needs	106.22	18.38	0.04	−0.04	−0.05	−0.05	0.46**	0.48**	0.48**	0.48**	0.95**	0.93**	0.95**	1	
13. Well-being	37.50	6.74	−0.09*	0.03	0.03	−0.05	0.77**	0.80**	0.80**	0.80**	0.56**	0.43**	0.59**	0.57**	1

*N* = 513; gender (M = 1, F = 2). *p < 0.05, **p < 0.01.

This study employed Baron and Kenny’s (1986) framework for mediation analysis, which specifies the intermediate variables to be examined, to investigate whether basic psychology needs mediate the relationship between aesthetic education and psychological well-being. Baron and Kenny’s (1986) mediation framework was selected for its well-established utility in examining intermediate variables within psychological research. This approach is particularly suited to explore the mediating role of basic psychological needs in the relationship between aesthetic education and psychological well-being. The model’s simplicity, robustness, and widespread application in similar studies ensure its reliability for testing the proposed mediation hypothesis. The analysis was conducted in three steps.

Step 1: The correlation between aesthetic education and well-being was tested. A significant positive relationship was found (*β* = 0.80, *p* < 0.01), indicating that aesthetic education is strongly associated with psychological well-being. The effect size in this step suggests a very large relationship, meaning that changes in aesthetic education have a substantial impact on students’ psychological well-being. In educational contexts, this indicates that fostering aesthetic education can play a crucial role in enhancing students’ mental health and emotional well-being.

Step 2: Aesthetic education was found to be a significant predictor of basic psychological needs (*β* = 0.59, *p* < 0.01), supporting the idea that aesthetic education influences the fulfillment of basic psychological needs. The medium-sized effect (*β* = 0.59) suggests that aesthetic education helps satisfy students’ intrinsic psychological needs such as autonomy, competence, and relatedness, which are fundamental to overall well-being. This finding underlines the importance of integrating aesthetic education into the curriculum as a means to fulfill psychological needs, which can have long-term benefits on students’ emotional development.

Step 3: Aesthetic education and basic psychology needs were simultaneously included in the regression model to test for mediation. The results showed that after controlling for basic psychological needs, aesthetic education continued to predict well-being (*β* = 0.31, *p* < 0.01); however, but with a substantial decrease between *β* = 0.80, *p* < 0.01 to *β* = 0.31, *p* < 0.01 suggesting that basic psychological needs partially mediate the relationship between aesthetic education and psychological well-being. This drop in the effect size from a large effect (*β* = 0.80) to a moderate effect (*β* = 0.31) indicates that while aesthetic education has a direct influence on psychological well-being, much of this effect is channeled through the satisfaction of basic psychological needs. This partial mediation suggests that interventions aimed at improving students’ well-being through aesthetic education should focus on fostering the fulfillment of psychological needs as a key mechanism. These results are summarized in Table 4, and Hypothesis 2 was partially supported.

**Table 4:** The mediating effects of basic psychology needs on aesthetic education and psychological well-being.

Variables	Step 1 (Well-being)		Step 2 (Basic psychology needs)		Step 3 (Well-being)	
	*β*	Sig.	*β*	Sig.	*β*	Sig.
Aesthetic education	0.80**	0.00**	0.59**	0.00**	0.31**	0.00**
Basic psychology needs					0.09**	0.00**
*F* value	900.10*		149.24**		551.61**	
Sig.	0.00**		0.00**		0.01**	
Adjusted *R*^2^	0.64		0.23		0.68	
SE	4.06		16.18		3.80	
Df	512		512		512	

*N* = 513. *p < 0.05, **p < 0.01.

In practical terms, these findings suggest that policymakers and educators should consider the substantial role of aesthetic education in promoting students’ mental health and well-being. The moderate effect size for mediation (*β* = 0.31) highlights the importance of psychological needs satisfaction as a pathway through which aesthetic education enhances well-being. Future educational policies could focus on integrating aesthetic education programs that address these needs to maximize their impact on students’ overall development.

In addition, this study employed hierarchical regression analysis (Aiken et al., 1991) to examine the moderating effect of age on the relationship between aesthetic education and psychological well-being. Hierarchical regression analysis is an effective technique for handling categorical moderator variables, enabling the assessment of interaction effects between aesthetic education and psychological well-being across different subgroups. This approach provides a comprehensive understanding of how age and gender differentially influence the relationship between these variables. Given that age is a categorical moderator, the sample was divided into three groups: 18–20 years old, 21–22 years old, and 23–25 years old. Separate regression analyses were conducted for each group to assess the impact of aesthetic education on psychological well-being. The moderating effect was considered significant if the differences in regression coefficients across the groups were statistically significant. The results of these analyses are presented in Table 5.

**Table 5:** The moderating effects of age on aesthetic education and psychological well-being.

Age		*B*	*t*	Sig.	*F*	df
Age 18–20	Constant	12.407	12.946	0.000	711.953	411
	AE	0.360	26.682	0.000		
*R*^2^ = 0.635 (Adjusted *R*^2^ = 0.634)						
Age 21–22	Constant	12.407	6.362	0.000	177.861	96
	AE	0.364	24.829	0.000		
*R*^2^ = 0.652 (Adjusted *R*^2^ = 0.648)						
Age 23–25	Constant	20.460	1.797	0.000	2.416	2
	AE	0.242	1.554	0.000		
*R*^2^ = 0.707 (Adjusted *R*^2^ = 0.415)						

*N* = 513. *p < 0.05, **p < 0.01.

For the 18–20 years old group, aesthetic education significantly predicted psychological well-being, with a standardized regression coefficient of 0.360 (*p* < 0.01). This indicates a moderate effect size, suggesting that aesthetic education has a meaningful impact on the psychological well-being of younger students. Given the developmental stage of this age group, the moderate effect size reflects the importance of aesthetic education in shaping emotional well-being and personal development during early adulthood. The practical implication of this finding is that incorporating aesthetic education into curricula for younger students can play a pivotal role in fostering their emotional resilience and overall psychological health.

For the 21–22 years old group, aesthetic education significantly predicted psychological well-being, with a standardized regression coefficient of 0.364 (*p* < 0.01). This effect size is also moderate, further reinforcing the notion that aesthetic education positively influences the psychological well-being of university students. As students in this age group are more likely to be dealing with academic stress and life transitions, the moderate effect size emphasizes the potential of aesthetic education to serve as a supportive tool in alleviating stress and enhancing emotional well-being during this critical period of personal and academic growth.

For the 23–25 years old group, aesthetic education significantly predicted psychological well-being, with a standardized regression coefficient of 0.242 (*p* < 0.01). This effect size is small, but it still indicates a statistically significant relationship between aesthetic education and psychological well-being. The smaller effect size in this group could suggest that as students grow older and accumulate more life experiences, the direct impact of aesthetic education on their psychological well-being may become less pronounced. However, even with a small effect size, the positive influence of aesthetic education on well-being highlights its ongoing value, suggesting that it can still play a supportive role in the well-being of older students, particularly in fostering emotional stability and creative expression.

Overall, these findings suggest that age moderates the impact of aesthetic education on psychological well-being, with younger students experiencing a stronger effect. The moderate effect sizes observed in the 18–22 years old groups imply that aesthetic education can have a substantial impact on their psychological development and well-being. These results have practical implications for educational policies, indicating that aesthetic education should be prioritized in university curricula, particularly for younger students, to enhance their psychological well-being and emotional development.

The statistical power of this study was considered sufficient due to the large sample size (513 participants), which provided robust estimates of the effects. The expected effect sizes were moderate to large, suggesting that the findings are meaningful and relevant for the context of this study. Future studies may consider using longitudinal designs or experimental methods to further examine causal relationships.

**Figure 1:**
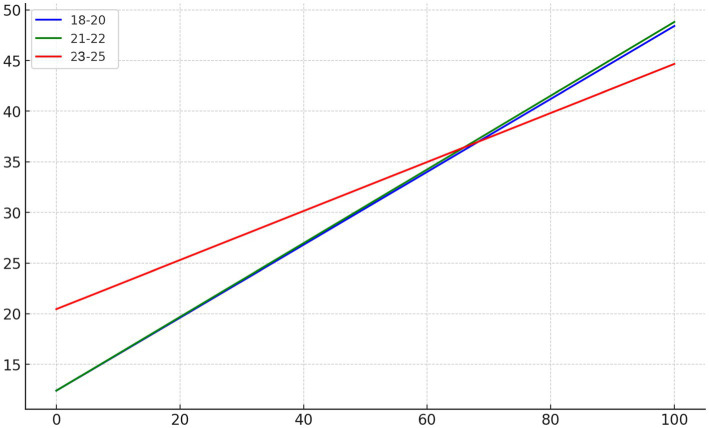
The moderating effect of age.

Based on the results of the regression equations, an interaction plot was created to illustrate the moderating effect of age on the relationship between aesthetic education and psychological well-being among college students. In Figure 1, the blue line represents the 18–20 years old group, the green line represents the 21–22 years old group, and the red line represents the 23–25 years old group. The plot shows the impact of varying levels of participation in aesthetic education (*X*-axis) on psychological well-being scores (*Y*-axis) for different age groups. The interaction plot shows that the three lines intersect, which indicates that the interaction is significant.

From the Figure 1, it is evident that for undergraduate students aged 18–20 and 21–22, higher levels of participation in aesthetic education are associated with higher psychological well-being scores. The effect size for this relationship is considered medium to large, indicating that aesthetic education has a substantial and meaningful impact on the psychological well-being of younger students. The positive relationship is particularly important in the context of university students, as it suggests that aesthetic education can be a key contributor to enhancing mental health and emotional regulation during the formative years of higher education.

For graduate students aged 23–25, increased participation in aesthetic education also positively affects psychological well-being scores, but the rate of change is slightly lower than that for undergraduates aged 18–20 and 21–22. The effect size for this group is smaller, indicating that while aesthetic education still has a beneficial impact, the magnitude of this effect is less pronounced in older students. This may be due to differences in emotional maturity, life experiences, or the academic pressures faced by graduate students, which could reduce the overall sensitivity to aesthetic education in comparison to younger students.

The interaction plot helps to visually represent the differences in the relationship between participation in aesthetic education and psychological well-being scores across different age groups. These findings suggest that age moderates the effect of aesthetic education on psychological well-being, with younger students showing a stronger response to aesthetic engagement. The varying effect sizes across age groups imply that interventions aimed at enhancing psychological well-being through aesthetic education may need to be tailored to the specific needs and characteristics of different student populations.

Based on the regression analysis and the interaction plot, Hypothesis 3 is supported, indicating that age plays a significant role in moderating the impact of aesthetic education on psychological well-being. These results suggest that the design of aesthetic education programs in universities could benefit from considering age-specific factors to maximize their positive impact on students’ mental health and overall well-being. In practical terms, this could inform policies and educational practices aimed at integrating aesthetic education into the curriculum in ways that are most effective for each age group.

Next, this study used hierarchical regression analysis (Aiken et al., 1991) to examine the moderating effect of gender on the relationship between aesthetic education and psychological well-being. Since gender is a categorical variable, the sample was divided into male and female groups, and separate regression analyses were conducted to assess the impact of aesthetic education on psychological well-being for each group. If the differences in regression coefficients across the groups were statistically significant, the moderating effect would be considered significant. The results are presented in Table 6.

**Table 6:** The moderating effects of gender on aesthetic education and psychological well-being.

Gender		*B*	*t*	Sig.	*F*	df
Male	Constant	11.633	6.924	0.000	270.742	128
	AE	0.379	16.454	0.000		
*R*^2^ = 0.679 (Adjusted *R*^2^ = 0.676)						
Female	Constant	12.929	12.985	0.000	616.492	381
	AE	0.351	24.829	0.000		
*R*^2^ = 0.618 (Adjusted *R*^2^ = 0.617)						

*N* = 513. *p < 0.05, **p < 0.01.

For the male group, aesthetic education significantly predicted psychological well-being, with a standardized regression coefficient of 0.379 (*p* < 0.01). This effect size is considered medium, indicating a relatively strong positive impact of aesthetic education on the psychological well-being of male students. Given that male students may face challenges in emotional expression and emotional regulation, this medium effect size highlights the potential of aesthetic education as a tool for enhancing psychological well-being, particularly in fostering self-expression and emotional regulation.

For the female group, aesthetic education also significantly predicted psychological well-being, with a standardized regression coefficient of 0.351 (*p* < 0.01). Although this effect size is slightly lower than that for the male group, it still falls within the medium range, suggesting that aesthetic education has a considerable positive impact on the psychological well-being of female students. Female students may be more open to emotional expression and social interaction, so aesthetic education has a direct and positive influence on their psychological health. While the effect size for the female group is slightly lower than that for the male group, this finding emphasizes the general value of aesthetic education for improving psychological well-being across genders.

Overall, gender played a moderating role in the relationship between aesthetic education and psychological well-being, with both male and female groups showing medium effect sizes. This suggests that the impact of aesthetic education on psychological well-being is similarly strong across genders. These findings have practical implications for educational practices, indicating that aesthetic education should be widely integrated into students’ academic lives as a tool to enhance psychological well-being and emotional development, regardless of gender.

**Figure 2:**
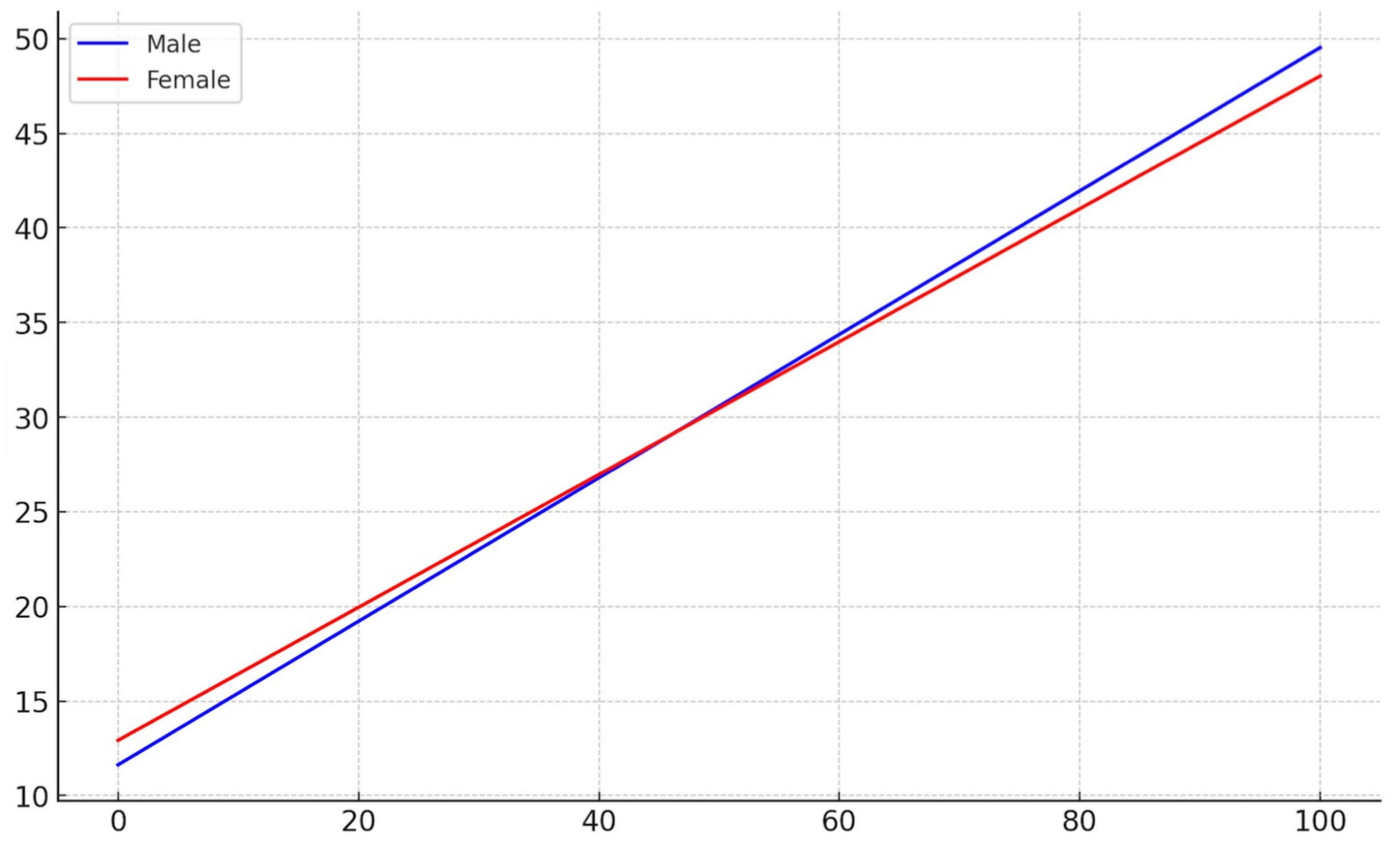
The moderating effect of gender.

Based on the results of the regression equations, an interaction plot was drawn to illustrate the moderating effect of gender on the relationship between aesthetic education and psychological well-being among college students. Figure 2 shows this effect, where the blue line represents males and the red line represents females. The plot demonstrates the impact of varying levels of participation in aesthetic education (*X*-axis) on psychological well-being scores (*Y*-axis) for different genders. The interaction plot shows that the two lines intersect, which indicates that the interaction is significant.

From the Figure 2, it can be observed that for male college students, higher levels of participation in aesthetic education correspond to higher psychological well-being scores. This relationship is characterized by a medium to large effect size, indicating that aesthetic education has a significant positive impact on male students’ psychological well-being. The magnitude of this effect suggests that aesthetic education could serve as a valuable tool for improving emotional well-being, especially for male students in higher education settings.

For female college students, increased participation in aesthetic education also positively affects psychological well-being scores, but the rate of change is slightly lower than that for males. This smaller effect size for females, though still significant, implies that other factors—such as social, cultural, or psychological influences—might moderate the relationship between aesthetic education and well-being for female students. This difference in effect sizes across genders could suggest that the impact of aesthetic education on psychological well-being might be more pronounced for male students, or that the benefits for female students might be influenced by additional variables.

This figure helps to visually display the gender-based differences in the relationship between aesthetic education and psychological well-being. These findings indicate that gender plays a moderating role in how aesthetic education impacts psychological well-being, with male students showing a stronger response to aesthetic education in terms of emotional and psychological benefits.

Based on the regression analysis and the interaction plot, we can conclude that Hypothesis 4 is supported, suggesting that gender moderates the relationship between aesthetic education and psychological well-being. These results have practical implications for designing gender-sensitive educational programs, which may need to take into account the differing ways in which aesthetic education influences male and female students. Understanding these gender-based differences can help educators tailor interventions to maximize their impact on student well-being across genders.

## 4 Discussion

In the field of education, it is well-established established that aesthetic education exerts a positive influence on various psychological outcomes in college students, including emotions, psychological well-being, and other significant outcome variables such as creativity and turnover behavior. The present study aimed to investigate the impact of aesthetic education on the psychological well-being of university students and developed a model to elucidate the underlying mechanisms through which aesthetic education affects psychological well-being in a university setting. Our findings indicate that basic psychological needs play a mediating role in the relationship between aesthetic education and psychological well-being. Furthermore, we discovered that age and gender act as moderating factor, influencing the strength and nature of the relationship between aesthetic education and psychological well-being. These results offer valuable insights into how educational contexts and aesthetic education can be strategically utilized to enhance psychological well-being of college students.

### 4.1 Theoretical contribution

This study makes several key theoretical, as outlined below:

Firstly, the study establishes that aesthetic education significantly and positively predicts the psychological well-being of college students. This findings aligns with previous research, which has emphasized the role of aesthetic education in promoting psychological health. Jin and Ye (2022) noted that art serves as a powerful medium for expressing human ideals, culture, identity, emotions, and societal experiences. Art, in particular, is recognized as a central component of aesthetic education (Ma et al., 2021), engages not only intellectual faculties but also the senses, emotions, and imagination. By facilitating holistic development beyond traditional academic measures, aesthetic education contributes significantly to individuals’ psychological well-being. Furthermore, recent studies in musical development, particularly in early childhood, have benefited from interdisciplinary insights drawn from educational theory, psychological well-being, and neuroscience (Lee and Chao, 2023). These perspectives underscore the broader impact of aesthetic education on emotional and mental health. Our findings extend this body of literature by demonstrating that aesthetic education is a robust predictor of psychological well-being among university students, providing valuable insights for the design and enhancement of university curricula. Davies et al. (2012) categorize aesthetic education into five broad domains: performing arts (including music, dance, theater, singing, and film), visual arts (such as design, craft, painting, photography, sculpture, and textiles), literature (comprising writing, reading, and attending literary events), and culture (encompassing visits to museums, galleries, art exhibitions, concerts, theater, and community events). Aesthetic activities within the university setting are inherently complex and multimodal, incorporating various elements known to promote health and psychological well-being among college students (Craig et al., 2008). As such, expanding the variety of aesthetic education courses and activities offered at university can significantly enrich students’ aesthetic experiences and foster improved psychological well-being.

Secondly, the research findings underscore the significant role of aesthetic education in enhancing the psychological well-being of college students, primarily through its impact on the satisfaction of basic psychological needs. The study confirms that aesthetic education positively influences students’ psychological well-being by facilitating the fulfillment of these needs. This mediation effect illustrates how engagement in aesthetic activities fosters creativity, self-expression, and an appreciation of beauty, thereby enhancing emotional resilience and overall fulfillment among students. These findings highlight the importance of incorporating aesthetic education into university curricula as a strategic approach to supporting students’ mental health. By expanding opportunities for aesthetic education, universities can create environments that not only stimulate cognitive development but also nurture emotional well-being. Such initiatives are crucial in addressing common challenges such as stress and anxiety, which are prevalent among students, thereby fostering a more supportive and conducive learning atmosphere. In summary, the research establishes clear causal pathways linking aesthetic education, the satisfaction of psychological needs, and enhancement of psychological well-being among college students. These insights advocate for the continued development and integration of aesthetic education programs within higher education institutions, with the aim of promoting holistic student development.

Thirdly, this study investigates the moderating effects of age and gender on the relationship between aesthetic education and psychological well-being among college students. Our findings demonstrate that both age and gender significantly influence how aesthetic education impacts psychological health. The hierarchical regression analyses reveal that aesthetic education significantly predicts psychological well-being across various age groups and genders, although the strength of this effect varies. Specifically, aesthetic education has the most pronounced positive impact on younger students (ages 18–22), enhancing their psychological well-being to a greater extent than for older students (ages 23–25). This suggests that in the early stages of university life, when students are adjusting to new environments and experiences, aesthetic education provides crucial emotional and cognitive support. For older students, while the benefits of aesthetic education remain positive, they appear to be somewhat attenuated, likely due to the different developmental and academic challenges encountered at this stage. Gender differences also emerged as significant, with female students reported higher levels of engagement in aesthetic activities, which correspond to greater improvements in emotional intelligence and stress management. Conversely, male students exhibited enhanced creativity and problem-solving abilities, benefiting from aesthetic education in distinct ways. These findings highlight the importance of tailoring aesthetic education programs to the unique needs of male and female students to optimize their psychological benefits.

### 4.2 Practical contribution

The implications of these findings suggest important considerations for educational policy and practice. While the study provides valuable insights into the relationship between aesthetic education and psychological well-being, the practical contributions should be viewed within the scope of this research. Universities may benefit from integrating more targeted and age-appropriate aesthetic education programs into their curricula. For younger students, programs focusing on emotional expression and social connectedness could be particularly beneficial. For older students, aesthetic education can be designed to support stress management and cognitive engagement, assisting them in dealing with the challenges of more advanced academic life. Additionally, gender-sensitive approaches may help ensure that both male and female students can benefit from aesthetic education. Tailoring art-based activities to diverse interests and strengths could create a more inclusive educational environment. However, further research is needed to fully understand how these programs can be implemented effectively across different contexts.

### 4.3 Limitations and future research suggestions

Although this study provides valuable insights into the relationship between aesthetic education and psychological well-being among university students, several limitations must be acknowledged. These limitations offer important directions for future research.

Frist, one limitation of this study is its cross-sectional design, which limits the ability to infer causal relationships. While this study highlights the association between aesthetic education and psychological well-being, it cannot confirm whether aesthetic education directly causes improvements in psychological well-being or if students with higher well-being are more likely to engage in aesthetic education. Future research should consider longitudinal designs to track changes over time, providing stronger evidence of causal relationships.

Second, this study employed convenience sampling, which, while practical, limits the generalizability of the findings. Participants were drawn from a single university in China, and as such, the results may not be applicable to other student populations, particularly those from different cultural or educational settings. Future research should aim for more diverse and representative samples, including students from various regions, universities, and countries, to enhance the external validity of the findings.

Third, data for this study were collected through self-report questionnaires, which may be subject to response biases such as social desirability or recall bias. Students may overreport their participation in aesthetic education or their psychological well-being due to perceived expectations or a desire to present themselves in a positive light. Future studies could incorporate objective measures, such as behavioral assessments or physiological indicators, to triangulate self-reported data and reduce bias.

Fourth, this study broadly defined aesthetic education, including various forms such as visual arts, music, literature, and dance. However, this broad definition did not explore the potential differential effects of each type of aesthetic education on psychological well-being. Future research could investigate how different forms of aesthetic education, such as music versus visual arts, may have distinct effects, and whether some forms are more effective at promoting specific aspects of psychological well-being.

Fifth, given the increasing reliance on online education, particularly following the COVID-19 pandemic, future research should explore the effects of aesthetic education delivered through digital platforms on students’ psychological well-being. Studies could examine whether the benefits of aesthetic education are amplified or diminished in virtual learning environments and how the absence of face-to-face interaction affects students’ well-being.

## 5 Conclusion

In conclusion, this study highlights the critical role of aesthetic education in enhancing psychological well-being of college students, with basic psychological needs acting as mediating factors and age and gender serving as significant moderating factors. By recognizing and addressing these factors, educational institutions can optimize the effectiveness of aesthetic education programs, thereby promoting the holistic development and well-being of students. The findings emphasize the importance of integrating tailored aesthetic education strategies to foster both emotional and cognitive growth, ultimately contributing to a supportive and enriching university experience.

## Data Availability

The raw data supporting the conclusions of this article will be made available by the authors, without undue reservation.
